# The relationship between perfectionism and self-esteem among trainee doctors

**DOI:** 10.1192/j.eurpsy.2024.1702

**Published:** 2024-08-27

**Authors:** S. Ellouze, N. Boussaid, A. Mellouli, M. Turki, E. Miledi, N. Halouani, J. Aloulou

**Affiliations:** Psychiatry “B” departments, Hedi Chaker University Hospital, Sfax, Tunisia

## Abstract

**Introduction:**

Perfectionism is often seen as a beneficial trait for trainee doctors since it means they have high standards and a drive for success. However, it demands a flawless level of performance regardless of one’s physical or mental health and well-being. Consequently, perfectionism is now viewed from a different perspective.

**Objectives:**

We aimed to study the impact of perfectionism among doctors in training on their self-esteem.

**Methods:**

We conducted a cross-sectional descriptive and analytical study among trainee doctors. The following psychometric instruments were used: The “Work Addiction Risk Test” and the “Rosenberg Self-Esteem Scale”.

**Results:**

We included 99 doctors in training. Their mean age was 27.6± 2.2 years. The sex ratio (♂/♀) was 0.33. Most participants were single (63.6%) and of middle socioeconomic level (86%). Tobacco use was reported in 6.1% of cases, and alcohol use in 4.1% of cases. Among all participants, 58 % had low or very low self-esteem. The mean score of Rosenberg Self-Esteem Scale was 30±5.13 and the mean score of the big three perfectionism scale short form was 41.11±13. Higher self-esteem scores was significantly associated with lower self-critical perfectionism scores.

**Conclusions:**

Our results point to the negative impact of self-critical perfectionism on self-esteem. In fact, perfectionism can hold you back, both personally and professionally. Perfectionists see their own self-worth as tied to what they achieve, and they believe that others judge them on this as well. They can never live up to the standards they set for themselves, and this can lead to a downward spiral of self-criticism and blame.

**Disclosure of Interest:**

None Declared

